# Click Chemistry in Polymersome Technology

**DOI:** 10.3390/ph17060747

**Published:** 2024-06-06

**Authors:** Nuno M. Saraiva, Ana Alves, Paulo C. Costa, Marta Correia-da-Silva

**Affiliations:** 1 LQOF—Laboratory of Organic and Pharmaceutical Chemistry, Department of Chemical Sciences, Faculty of Pharmacy, University of Porto, Rua Jorge de Viterbo Ferreira 228, 4050-313 Porto, Portugal; nunommsaraiva@gmail.com; 2 CIIMAR—Interdisciplinary Center of Marine and Environmental Research, University of Porto, Terminal dos Cruzeiros do Porto de Leixões, Avenida General Norton de Matos, S/N, 4450-208 Matosinhos, Portugal; 3 UCIBIO—Applied Molecular Biosciences Unit, MedTech-Laboratory of Pharmaceutical Technology, Faculty of Pharmacy, University of Porto, Rua Jorge de Viterbo Ferreira 228, 4050-313 Porto, Portugal; anadaniela92@hotmail.com (A.A.); pccosta@ff.up.pt (P.C.C.); 4 Associate Laboratory i4HB, Institute for Health and Bioeconomy, Faculty of Pharmacy, University of Porto, Rua Jorge de Viterbo Ferreira 228, 4050-313 Porto, Portugal

**Keywords:** polymersome, click chemistry, CuAAC, surface modification, copolymer synthesis

## Abstract

Polymersomes, self-assembled nanoparticles composed of amphiphilic block copolymers, have emerged as promising versatile nanovesicles with various applications, such as drug delivery, medical imaging, and diagnostics. The integration of click chemistry reactions, specifically the copper [I]-catalysed azide–alkyne cycloaddition (CuAAC), has greatly expanded the functionalisation and bioconjugation capabilities of polymersomes and new drugs, being this synergistic combination explored in this review. It also provides up-to-date examples of previous incorporations of click-compatible moieties (azide and alkyne functional groups) into polymer building blocks, enabling the “click” attachment of various functional groups and ligands, delving into the diverse range of click reactions that have been reported and employed for polymersome copolymer synthesis and the modification of polymersome surfaces, including ligand conjugation and surface modification. Overall, this review explores the current state-of-the-art of the combinatory usage, in recent years, of polymersomes with the click chemistry reaction, highlighting examples of studies of their synthesis and functionalisation strategies.

## 1. Introduction

### 1.1. Click Chemistry

The 2022 Nobel Prize in chemistry was awarded to Carolyn Ruth Bertozzi, Morten Meldal, and Karl Barry Sharpless [1] and brought a new window of possibilities to the pharmaceutical and material industries. Sharpless and co-workers [2] enormously impacted chemistry philosophy by discovering “click” reactions. Click chemistry is a class of nearly perfect chemical reactions that are effective in terms of atom economy, stereospecificity, wide scope, and almost all properties that today are called the green chemistry principles. The reaction is enormously selective since it only occurs when azide and alkyl groups are present.

**Figure 1:**
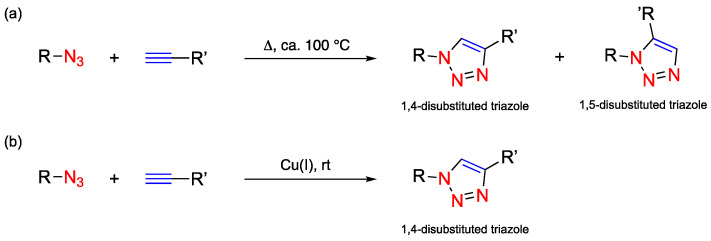
Schematic representation of Huisgen 1,3-dipolar cycloaddition variants. In (**a**) the classical thermal azide–alkyne cycloaddition (AAC) and (**b**) the copper-catalysed azide–alkyne cycloaddition (CuAAC). rt, room temperature.

The copper-catalysed azide–alkyne cycloaddition (CuAAC) is a variant of the classical thermal Huisgen 1,3-dipolar cycloaddition and was described by Sharpless as the ‘cream of the crop’ of click chemistry [3,4]. By using copper (CuSO_4_) and sodium ascorbate, the energy necessary for the activation barrier is decreased significantly, making the reaction possible to proceed at room temperature and in aqueous or organic solvents, leading to a 1,4-disubstituted triazole (Figure 1) [5].

These Nobel laureates also impacted chemical and biorthogonal chemistry philosophy, which can be briefly defined as the “chemical reaction that can occur inside of living systems without interfering with native biochemical processes”. With the discovery of “click” reactions, the need to proceed with biorthogonal reactions under conditions that would not harm and preserve such biological systems was solved. Bertozzi’s group coined this term, taking inspiration from the mathematical term “orthogonality”—two variables that vary and coexist independently from one another. In a broad sense, this class of chemistry allows for the use of normal organic synthesis techniques to be applied to complex living systems, such as cells [6,7,8].

Overall, to be considered a ‘click’ reaction, several characteristics similar to green chemistry principles must be satisfied [2]. Theoretically, there is neither the need to use protective groups in click reaction synthesis nor the use of extensive chromatographic purification methods [9,10,11]. This class of reactions had already impacted the scientific world in a broad sense, given its properties [10,11,12,13].

### 1.2. Polymersomes

**Figure 2:**
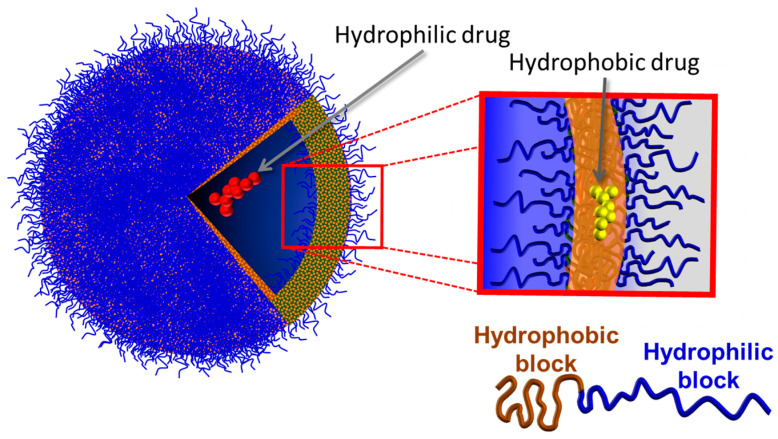
Schematic representation of a polymersome.

Polymersomes (PMs) are spherical and hollow nanosystems composed of amphiphilic copolymers that can encapsulate hydrophilic and hydrophobic drugs, individually or at the same time, and go from 100 nm to a few μm in diameter [14] (Figure 2).

Discher’s group was the first to use the term ‘polymersome’ in 1999 when they developed and described a polymeric structure composed of polyethyleneglycol (PEG)–polyethylethylene (PEE) [15]. A study has revealed that the synthetic polymer length is up to 10 times larger than the phospholipid acyl chain [15,16]. Other studies have also contributed to highlighting that the thickness of polymersomes (2–30 nm) [15,17,18,19,20,21] is more significant than the liposome (3–5 nm) [18,19], providing more stability and protection against mechanical/chemical shear, giving polymersomes more stability, more content retention [22], superior functionalisation, high choice of drug encapsulation, bioavailability, biodegradability, and changeable mechanical properties, applications, and cargo release induced by stimuli compared to other drug delivery systems (DDSs) (Table 1). Therefore, polymersomes have a wider range of applications and are an excellent solution and a modern candidate for DDSs [23,24].

Despite their excellent attributes, polymersome research still faces some challenges and limitations. Achieving precise control over polymersome size and membrane properties (such as permeability) and the scalability for large-scale production remains a challenge [35]. Maintaining stability during storage and delivery, performing efficient encapsulation of hydrophilic and hydrophobic cargos, and achieving long-term stability in biological environments are ongoing issues. Moreover, optimising polymersome targeting, biodistribution, and clearance properties requires further investigation [36,37].

**Figure 3:**
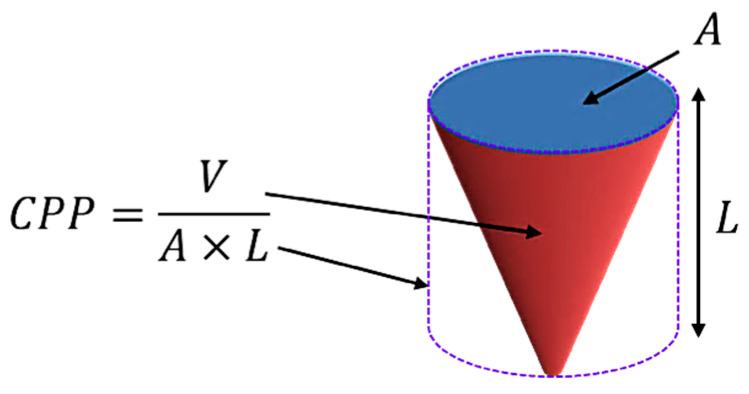
Critical packing parameter equation concerning the membrane disposition: where *V* is the volume of the hydrophobic part, *A* is the area of the hydrophilic fraction, and *L* is the length of the chain.

Different structures can be formed depending on the type of copolymers used and the hydrophobic fraction. The interfacial tension between the hydrophobic part and the water auto-modulates the copolymers, creating kinetically stable vesicles. By reducing the size of this fraction, the result is the development of cylindrical micelles rather than spherical micelles, and the continuous reduction will lead to the development of spherical vesicles and, essentially, polymersomes [38]. Each copolymer block’s physical properties will define how these polymers are “packed”, and by using the critical packing parameter (*CPP*), the most probable type of structure achieved can predicted (Figure 3), where if the 1 > *CPP* > 1/2, polymersomes are formed.

In this equation, *V* is the volume of the hydrophobic block, *A* is the interfacial area per molecule, and *L* is the hydrophobic block length [39,40,41]. As a result, if the value of *CPP* is equal to one, we will see planar lamellae forming (Table 2); if 1/2 > *CPP* > 1/3, we can observe cylindrical micelles; when *p* is smaller than 1/3, we can observe spherical micelles. If, by any chance, the value of *CPP* is higher than 1, inverted structures are expected to be present [39,41,42].

This review summarises the state-of-the-art on polymersome copolymer synthesis and functionalisation using 1,4-disubstituted 1,2,3-triazole as the central linker between polymer–polymer and polymer–ligand by click chemistry.

## 2. Click Chemistry in Copolymer Synthesis

One polymer sequence is called a “block”, and copolymers are defined as two or more sets of polymers connected to form an amphiphilic macromolecule [43]. Given its molecular design, the copolymer on the membrane itself can have different conformations, such as diblock, triblock, or multiblock [44,45,46,47,48,49,50].

**Figure 4:**
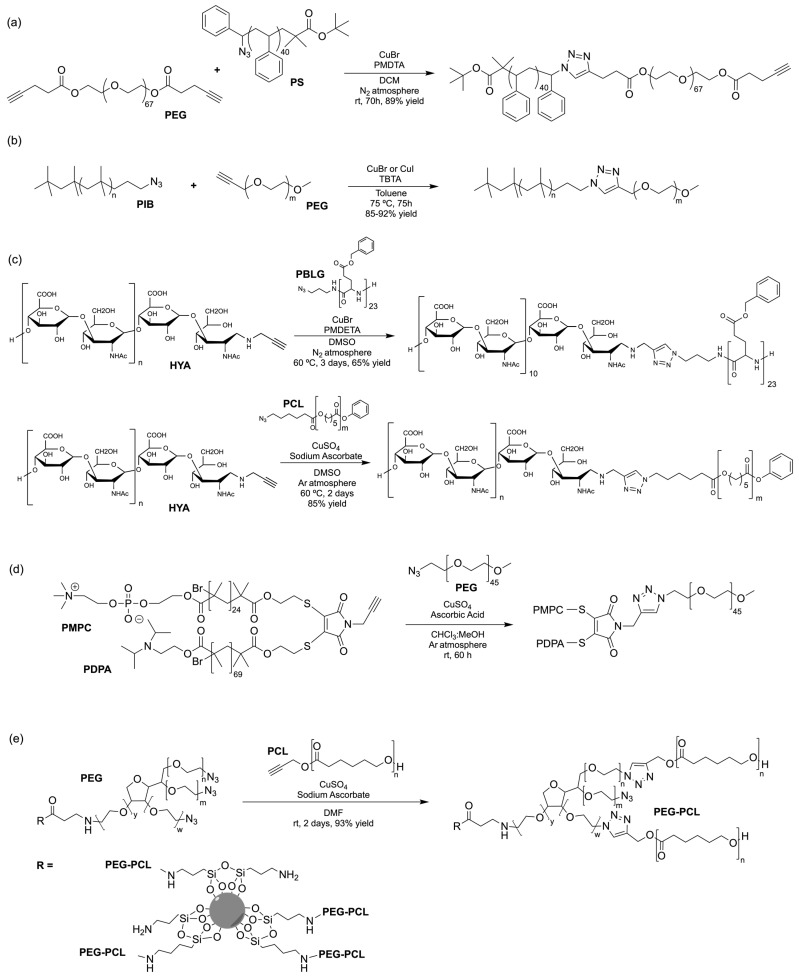
Compilations of copolymer synthesis. (**a**) Synthesis of PS-PEG diblock copolymer; (**b**) synthesis of PIB-PEG diblock copolymer; (**c**) synthesis of HYA–derivate diblock copolymers. The first: HYA-PBLG copolymer synthesis. The second: HYA-PCL copolymer synthesis; (**d**) synthesis of a PEG-PMPC-PDPA triblock miktoarm copolymer; (**e**) synthesis of a PEG-PCL diblock copolymer. PS: polystyrene; PEG: polyethyleneglycol; PIB: polyisobutylene; HYA: hyaluronan; PCL: polycaprolactone; PBLG: poly *γ*-benzyl glutamate; PMPC: poly-2-(methacryloyloxy)ethyl choline phosphate; PDPA: poly-2-(diisopropylamino)ethyl methacrylate.

The CuAAC has been previously used to connect the different blocks. It was proven in 2005 that the synthesis of copolymers via the 1,3-dipolar cycloaddition of terminal azide and alkyne functionalised polymers was possible and provided good yields [51]. Years after, in 2008 and 2009, this strategy was integrated into polymersome’s copolymer block synthesis. The first publication reports the aggregation of a diblock copolymer composed of polystyrene (PS)-PEG synthesised by CuAAC. Briefly, the aim was to functionalise a PS–poly[isocyanoalanine(2-thiophen-3-yl-ethyl)amide (PIAT) polymersome with an enzyme. A diacetylene-functionalised PEG chain allowed for cycloaddition with the PS–azide copolymer (Figure 4a). After polymersome assembly, the free acetylene group reacted with an azide enzyme [52,53].

The CuAAC reaction was also used by Binder et al. to synthesise a PEG–polyisobutylene (PIB) diblock copolymer in 2008 [54] (Figure 4b), by Kumar et al. for the synthesis of hyaluronan (HYA)–poly *γ*-benzyl glutamate (PBLG) in 2009 [55] (Figure 4c), and by Shahriari et al. for the synthesis of HYA–polycaprolactone (PCL) in 2021 [56] (Figure 4c).

Besides the typical diblock copolymer, another two groups focused on synthesizing and connecting triblock copolymers into a miktoarm shape. In 2012, Yin et al. [57] reported the synthesis of PEG–(poly His)_2_ 3-miktoarm, mimicking a phospholipid structure assembled in the aqueous phase into polymersomes that presented low cytotoxicity and pH sensitiveness. This particular copolymer was described as a pH-dependent drug release system. Later that decade, Battaglia et al. [58] successfully synthesised a 3-miktoarm copolymer with three distinct arms connected by a dibromo-N-propargyl-maleimide motif. Poly-2-(diisopropylamino)ethyl methacrylate (PDPA) and poly-2-(methacryloyloxy)ethyl choline phosphate (PMPC) were synthesised with a sulphide moiety that was later attached to the maleimide central block. The PEG block attachment was the last step using the CuAAC reaction (Figure 4d). The resulting miktoarm copolymer was able to induce the assembly of polymersomes.

Another study in 2018 by Khoee et al. [59] demonstrated a complex polymersome structure that combines the advantages of magnetite (Fe_3_O_4_) nanoparticles and a three-layer copolymer. This structure comprises a PCL layer between two inner and outer PEGs, which were connected after the click reaction (Figure 4e) between the first azide–PEG moiety and the alkyne–PCL.

## 3. Click Chemistry in Polymersome Functionalisation

Polymersomes can be functionalised with various molecules or groups to introduce specific properties or functionalities to their structure. The functionalisation of polymersomes enables customisation and tailoring of their behaviour for specific biomedical applications [60]. The choice and combination of functionalisation strategies depend on the desired properties, targeted applications, and the compatibility of the functional molecules with the polymersome structure.

Surface modification can be conducted through various methods and reactions, namely, the inverse electron-demand Diels–Alder [61,62,63], thiol-ene chemistry [64,65,66], click chemistry [67,68,69], or Diels–Alder reactions [70,71,72]. However, this broad spectrum of reactions is not always possible, given their limitations in material functionalisation. The use of the CuAAC reaction on polymersome functionalisation is relatively new, and few different ligands have been reported to be attached to a polymersome outer surface by different reactions [73], and each one of them will be explored.

Firstly, for the CuAAC to be possible, it is necessary to have a pair of alkyne and azide functional groups. Usually, these groups are absent in the ligands and in the polymers to be connected, so the introduction of these groups needs to be accomplished first. Commonly, introducing an azide group takes place by a diazo-transfer reaction, allowing for the creation of an azide from a primary amine using the diazo-transfer agents. However, there is not always a presence of an amine to change to an azide functional group, so other methods and reactions are possible. Table 3 shows a compilation of reactions and conditions used for the introduction of an azide or an alkyl end group on copolymer synthesis and copolymer functionalisation.

**Figure 5:**
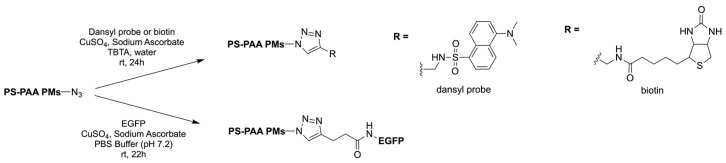
Functionalisation of PS-PAA diblock copolymer polymersomes with fluorescent dansyl probe, biotin ligands, and EGFP.

One of the first synergic uses of click reactions to functionalise polymersomes was described in 2007 by Opsteen et al. when PS–polyacrylic acid (PAA) copolymer was prepared by atom transfer radical polymerisation (ATRP). After the polymerisation, an azide functional group was placed on the PAA end chain (Table 3, entry 8), and after the polymersome self-assembly, the CuAAC reaction took place (Figure 5) to introduce a fluorescent dansyl probe, biotin ligands, and an enhanced green fluorescent protein (EGFP), previously alkylated (Table 3, entry 14) on the nanoparticle [78].

**Figure 6:**
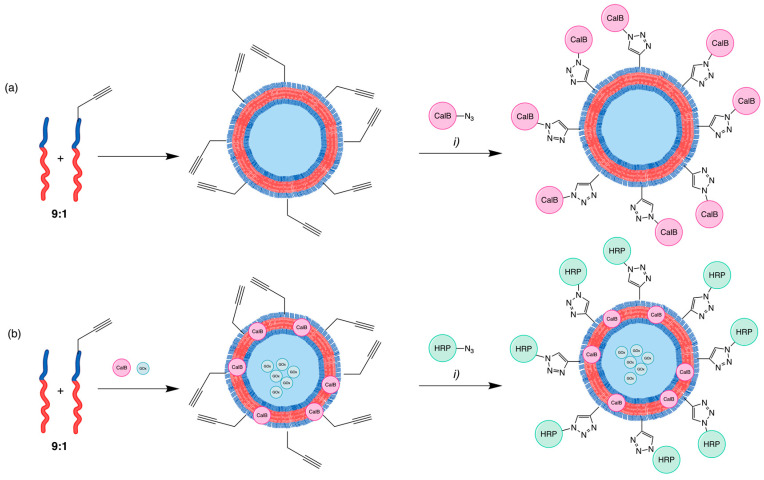
Functionalisation of PEG-PIAT and PEG-PS (9:1) diblock copolymer polymersomes with CalB (**a**) and HRP (**b**). (*i*) Azido-functionalised enzyme (2 eq.), CuSO_4_.5H_2_O, sodium ascorbate, bathophenanthroline ligand, phosphate buffer (pH 7.4), 4 °C, 60 h.

After this functionalisation report was published, some different approaches were studied. For example, in 2008 and 2009, van Dongen et al. [52,53] described two similar techniques using a PEG-PS copolymer with an alkyl end group on the PEG block (Table 3, entry 17) to introduce a *Candida antarctica* Lipase B (CalB) via CuAAC, after the polymersome assembly. This biohybrid polymersome showed enzymatic activity (Figure 6a). The second approach incorporated three enzymes into the polymersome’s structure: glucose oxidase (GOx) was put into the lumen of the polymersome. At the same time, CalB was contained inside the polymeric bilayer membrane, and a triazole connected horseradish peroxidase (HRP) to the polymersome surface (Figure 6b).

More studies about the functionalisation of polymersome surfaces were published. They englobe dendrimers [79,80,81], polysaccharides [77], peptides [74], anti-tumoral drugs [83], polymersome immobilisation [82], and the use of polymersomes as nanoreactors [75,76]. More detailed information about polymersome functionalisation ligands can be found in Table 4.

## 4. Structural Elucidation

### 4.1. Triazole and Azide–Alkyne Elucidation

It is essential to know how to proceed and synthesise these macromolecules and, more importantly, to know if we have successfully reached our goal, and that can be achieved by spectroscopic methods and structure elucidation studies.

Concerning the copper cycloaddition, the general approach used was proton or carbon nuclear magnetic resonance (^1^H/^13^C NMR) and Fourier transform infrared spectroscopy (FTIR). The study of Zhang et al. on a polylactide (PLA) polymer gives us a remarkable insight into these methodologies [84]. Other studies corroborate these values [85,86,87,88,89].

Overall, the presence of the azide group can easily be confirmed by the presence of an absorption band in 2090–2160 cm^−1^ (N=N=N stretching) in the IR spectrum. IR and NMR can confirm the presence of the alkyne. In the IR spectrum, an absorbance band at 2102–2129 cm^−1^ due to triple-bond CC stretching and another at 3273–3288 cm^−1^ due to H-C alkyne stretching can easily be detected. The presence of the alkyne group can also be confirmed by NMR by the presence of a triplet resonance signal around 2.5–3.3 ppm (-C≡C***H***) in the ^1^H NMR spectrum and by the presence of two signals around 77–78 ppm (-***C***≡***C***) in the ^13^C NMR spectrum. NMR is very useful to confirm the formation of the triazole by the presence of a singlet at 7.6–7.9 ppm (triazole ***H***) in the ^1^H NMR spectrum and two signals around 142–145 ppm and 123–128 ppm (triazole ***C=C***) in the ^13^C NMR spectrum.

### 4.2. Nanoparticle Assemble Elucidation

To confirm the stability and function of the polymersomes, it is essential to have a method to analyse and check their integrity status. Given the current characterisation methods used, the ones used on polymersomes include microscopy, light scattering, and gel permeation chromatography.

Microscopy methods are easy to use and provide specific and simple visualisations [90]. Visualisation of polymersomes is essential to assess their size, configuration, morphology, and homogeneity. Two types can be used: light and electron microscopy [41,91]. Polymersomes can be directly visualised on an aqueous dispersion for light microscopy, with no need for modifications for visualisation. However, it is only possible to see large-sized particles (diameter > 1 µm). In better resolution studies (diameter > 1 nm), it is possible to use electronic microscopy—scanning electron microscopy (SEM) or transmission electron microscopy (TEM)—with the disadvantage of requiring drying and staining of the sample to enhance the contrast.

By the techniques of light scattering, either dynamic (DLS) or static, also known as laser diffraction (LD), it is possible to measure the size of the particle, e.g., the diameter and the size distribution. With this, it is also possible to study the effect of the pH/temperature on the vesicle’s conformation, the critical aggregation concentration, and even the membrane disruption [92]. The DLS method can also determine the outer membrane’s zeta potential. These characterisation methods are easy, quick, and precise, requiring only data analysis over the complex surfactant system that the polymersomes are in [91]. Interestingly, some studies have previously described an increase of 50–100 nm in diameter of the polymersomes after their functionalisation via click chemistry [75,76,80]. Also, these assays by DLS described that polymersomes have a mean diameter between 100 and 200 nm [55,56,57,59,75,76,80,82], excluding the polymersomes with a glycosylated PE-PEG copolymer, which had a diameter of 25–50 µm [77].

Other methods using X-ray scattering, such as small-angle X-ray scattering (SAXS) and wide-angle X-ray scattering (WAXS), are being used to complement and provide more detailed information about structural characteristics. One specific neutron-scattering (SANS) was reported to be helpful in investigations of morphology, structure, copolymer self-assembly, and thermodynamic factors of the polymers [41]. Differential scanning calorimetry (DSC) is another technique capable of analysing the structure of the copolymers by thermal behaviour [93,94]. Studies on polymersomes’ copolymers have shown that the thermal behaviour is altered, either by the presence of an encapsulated drug [23,24] or by the presence of the triazole group [84].

Additionally, the use of gel permeation chromatography (GPC) can provide insights into the analysis of size distributions, characterisations of molecular weight distributions, and polymer quality control. A given size distribution can be used to analyse polymer clusters in the pre-gel state, which helps to determine the optimal conditions for the polymersome’s synthesis.

GPC can be used during the development, production, and quality control of polymersomes. This can help to ensure that the polymersomes meet the desired specifications and are safe for use [95,96].

## 5. Conclusions and Future Perspectives

Due to their unique properties and applications, polymersomes have earned some attention in recent years. These synthetic vesicles have demonstrated remarkable stability, biocompatibility, and tunability. They have been extensively explored in many biomedical and nanotechnology applications, including nanoparticle drug delivery.

The integration of click chemistry, particularly the copper(I)-catalysed azide–alkyne cycloaddition (CuAAC), into polymersome technology has opened up a plethora of possibilities for advancements in biomedical applications.

This review analysed both copolymer synthesis and copolymer functionalisation through the application of the CuAAC reaction—a powerful tool in various research fields, such as polymer and materials science, medicinal chemistry, chemical biology, and pharmaceutical sciences. The CuAAC reaction was used for the formation of copolymers between hydrophilic polymers PEG and HYA, as well as hydrophobic polymers PS, PIB, PBLG, PCL, PDPA, and PMPC. Several ligands were also introduced by CuAAC: enzymes (CalB, GOx, and HRP), polysaccharides (fucose and glucose), dendrons, peptides (GRGDSP, PR_b, and EGFP), dye probes, metallic complexes, an anti-tumoral drug, and an L-Proline catalyst. Also, this review describes the reactions, and their conditions, that were necessary to introduce the alkyl and azide functional groups in the building blocks, since these functional groups do not occur naturally in the majority of molecules.

By enabling rapid and precise synthesis, click chemistry can transform industries. By leveraging the precision and specificity of click reactions, it is possible to easily produce polymersomes with multiple ligands and functional groups, enhancing their targeting capabilities and therapeutic efficacy.

Key findings highlight the versatility and efficiency of click reactions, enabling precise control over molecular structures, the synthesis of complex molecules, and the development of functional materials. The advancements in click chemistry and polymersome technology are likely to have a profound impact on the pharmaceutical and material science industries. The principles of green chemistry inherent in click reactions align with the increasing demand for sustainable and environmentally friendly processes. Moreover, the ease of functionalisation and the ability to perform reactions under mild conditions make these methods highly attractive for large-scale production.

This review proved that ‘Click’ is essential in the design and synthesis of polymersome polymers, facilitating targeted drug delivery, controlled release systems, and other innovative applications.

## Figures and Tables

**Table 1:** Polymersomes (PMs) compared with other drug delivery systems (DDSs).

DDS	Disadvantages Compared to PMs
Liposomes	The thickness of the liposome (3–5 nm) provides less stability, and less retention of content [16].
Solid Liquid Nanoparticles	These particles have some disadvantages, such as the rapid loss of large quantities of drugs and the lack of controlled drug release [25,26].
Microemulsions	Less stable—they can be affected by temperature, pH, and other environmental factors and have lower encapsulation efficiencies [27].
Micelles	Reduced stability in the bloodstream, since the critical micellar concentration (CMC) can be reduced by blood dilution and the encapsulated drugs can leak out, minimizing drug circulation [28,29].
Dendrimers	Showed cytotoxicity [30].
Quantum Dots	Cytotoxicity of small semiconductor particles [31].
Carbon nanotubes	The process of production is expensive and lacks solubility in aqueous media [32].
Silver nanoparticles	Toxic effects on cells and organisms [33].
Golden nanoparticles	The methods used for the synthesis are expensive and can also use toxic ingredients. This makes it difficult to implement this recent technology in all the places where it could be useful [34].

**Table 2:** Packing formation and membrane conformation given the *CPP* value of amphiphilic membranes. The “Interface Formed” column represents phospholipid membranes, although polymersomes’ are similar.

Packing Formation	*CPP*	Interface Formed
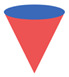	<1/3 (spherical) 1/3–1/2 (cylindric micelles)	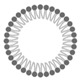
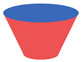	1/2–1 (Flexible lamellae, vesicles, polymersomes)	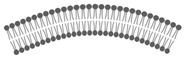
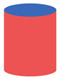	≈1 (Planar lamellae)	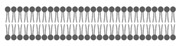
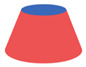	>1 (Inverted structures)	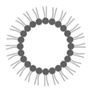

**Table 3:** Compilation of reactions used for azide–alkyl functional group introduction in copolymers for polymersomes’ formation.

Reactions Used for Azide–Alkyl Functional Group Introduction				
Entry	Transfer Azide–Alkyl	End Group	Reaction Conditions	Ref.
1	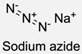	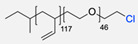	Argon atmosphere, DMF, 65 °C Undisclosed yield	[74]
2		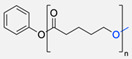	(i) TsCl, Et_3_N, DCM, rt (ii) NaN_3_, DMF, rt Undisclosed yield	[55,56]
3		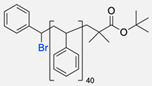	DMF, rt 89% yield	[52]
4		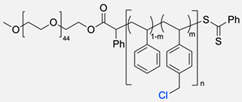	DMF, rt 66% yield	[75,76]
5		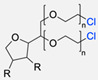	DMF, 120 °C (4 h) 99% yield	[59]
6		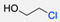	H_2_O, 80 °C (24 h) 80–90% yield	[77]
7		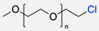	N_2_ atmosphere, DMF, rt (24 h) 85% yield	[57]
8	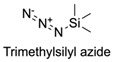	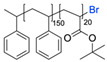	TBAF, THF, rt Undisclosed yield	[78]
9	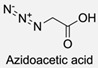	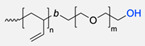	DCC, DMAP, DPTS, DCM, rt 95% yield	[79,80,81]
10	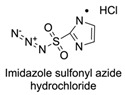	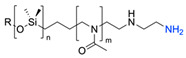	K_2_CO_3_, [Cu^(II)^SO_4_] *, H_2_O, rt Undisclosed yield	[82]
11	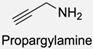	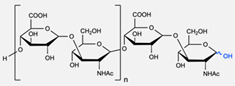	NaBH_3_CN, Acetate buffer, 50 °C (5 days) Quantitative yield	[55,56]
12		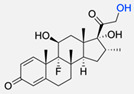	(i) MsCl, N_2_ atmosphere, Pyridine, rt (ii) N_2_ atmosphere, DMF, 65 °C (2 h) 62% yield	[83]
13		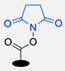	NaHCO_3_, rt (1.5 h) Undisclosed yield	[82]
14	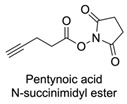	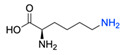	DCM, rt (2 h) 97% yield	[78]
15	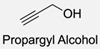	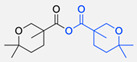	DMAP, DOWEX H^+^, MeOH, Pyridine 95% yield	[79,80,81]
16		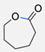	Sn(Oct)_2_, 100 °C (18 h) Undisclosed yield	[59]
17	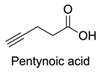	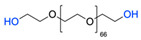	EDC, DMAP, DCM, rt 84% yield	[52,53]
18		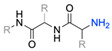	DIPEA, HBTU, DMF, rt Undisclosed yield	[74]
19	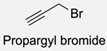	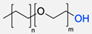	NaH, THF, N_2_ atmosphere, rt (2 h)–70 °C (6 h) 88% yield	[77]
20		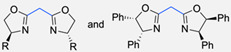	BuLi, THF, −10 °C (3 h) 66% yield	[75]
21		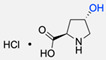	NaH, THF, 0 °C (2 h)–rt (18 h) 57% yield	[76]

* Optional use. DCC: N, N′-Dicyclohexylcarbodiimide; DCM: dichloromethane; DIPEA: N, N-Diisopropylethylamine; DMAP: 4-Dimethylaminopyridine; DMF: dimethylformamide; DPTS: 1,4-Dimethylpyridinium p-toluenesulfonate; EDC: 1-Ethyl-3-(3-dimethylaminopropyl)carbodiimide; HBTU: Hexafluoro-Phosphate Benzotriazole Tetramethyl Uronium; MsCl: methane sulfonylchloride; rt: room temperature; TBAF: tetra-n-butylammonium fluoride; THF: tetrahydrofuran; TsCl: toluenesulfonyl chloride.

**Table 4:** Details about functionalisation reactions, showcasing all different di- and triblock copolymers used, the ligand connected, and main achievements.

Hydrophilic Block Polymer	Hydrophobic Block Polymer	Ligand	Main Achievements	Ref.
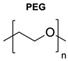	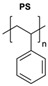	Enzymes: CalB, GOx, and HRP	Functionalised polymersomes increased the local concentration of enzymes, leading to higher reaction rates, making it possible to remove catalytical enzyme species in one single step.	[52,53]
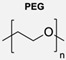	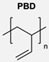	Peptides GRGDSP and PR_b	Polymersomes functionalised with the peptides were more effective in delivering doxorubicin to colon cancer cells than “naked” polymersomes. The functionalisation allowed for precise targeting, which is crucial for minimising off-target effects and maximising therapeutic efficacy.	[74]
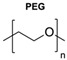	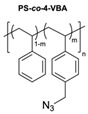	Copper-bis(oxazoline) complexes	The hydrophobic layer of polymersomes allowed for the immobilisation of the metal complex, making the reaction possible to occur in an aqueous media instead of an organic solvent.	[75]
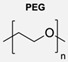	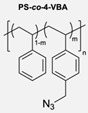	L-Proline catalyst	The hydrophobic layer of polymersomes allowed for the immobilisation of the catalyst, making the reaction possible to occur in an aqueous media instead of an organic solvent and therefore improving the yield, diastereoselectivity, and enantioselectivity.	[76]
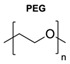	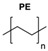	Sugars: Fucose and Glucose	The functionalisation of the polymersomes with D-glucoside allowed for better binding and affinity to their lectins (carbohydrate-binding proteins), proving to be a valuable strategy for targeted drug delivery.	[77]
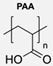	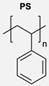	Fluorescent dansyl probe, biotin ligands, and EGFP	This work proved that functionalisation of the outer membrane of polymersomes is possible.	[78]
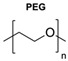	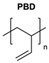	Dendrons	The dendritic architecture allowed for the conjugation of multiple functional groups, such as chromophores and biologically relevant ligands, increasing the versatility of polymersomes.	[79]
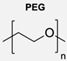	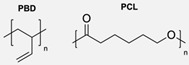	Dendrons	Surface functionalisation of polymersomes with dendritic groups offered a valuable framework for controlling their biological properties without significantly affecting their physical characteristics, such as size and stability.	[80]
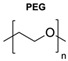	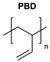	Dendrons	With this study, an ideal percentage for azide polymer in polymersome vesicles was determined. Also, it was determined that the presence of the dendron group did not alter the polymersome morphology.	[81]
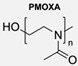	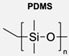	Dyes and PAN membranes	Immobilisation of polymersomes on a planar solid structure was shown to be possible for production, usage, and handling.	[82]
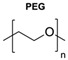	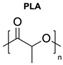	Dexamethasone	Dexamethasone-functionalised polymersomes proved to be more effective than “naked” particles in pancreatic cancer cells.	[83]

## Data Availability

Data sharing is not applicable.

## References

[1] (2022). The Nobel Prize in Chemistry 2022. https://www.nobelprize.org/prizes/chemistry/2022/press-release/.

[2] Kolb H.C., Finn M.G., Sharpless K.B. (2001). Click Chemistry: Diverse Chemical Function from a Few Good Reactions. Angew. Chem. Int. Ed..

[3] Rostovtsev V.V., Green L.G., Fokin V.V., Sharpless K.B. (2002). A Stepwise Huisgen Cycloaddition Process: Copper(I)-Catalyzed Regioselective “Ligation” of Azides and Terminal Alkynes. Angew. Chem. Int. Ed..

[4] Kondengadan S.M., Bansal S., Yang C., Liu D., Fultz Z., Wang B. (2023). Click chemistry and drug delivery: A bird’s-eye view. Acta Pharm. Sin. B.

[5] Devaraj N.K., Finn M.G. (2021). Introduction-Click Chemistry. Chem. Rev..

[6] Agard N.J., Baskin J.M., Prescher J.A., Lo A., Bertozzi C.R. (2006). A Comparative Study of Bioorthogonal Reactions with Azides. ACS Chem. Biol..

[7] Saxon E., Bertozzi C.R. (2000). Cell Surface Engineering by a Modified Staudinger Reaction. Science.

[8] Sletten E.M., Bertozzi C.R. (2011). From Mechanism to Mouse: A Tale of Two Bioorthogonal Reactions. Acc. Chem. Res..

[9] Le Droumaguet B., Velonia K. (2008). Click chemistry: A powerful tool to create polymer-based macromolecular chimeras. Macromol. Rapid Commun..

[10] Slavin S., Burns J., Haddleton D.M., Becer C.R. (2011). Synthesis of glycopolymers via click reactions. Eur. Polym. J..

[11] Takayama Y., Kusamori K., Nishikawa M. (2019). Click chemistry as a tool for cell engineering and drug delivery. Molecules.

[12] Bartenstein J.E., Robertson J., Battaglia G., Briscoe W.H. (2016). Stability of polymersomes prepared by size exclusion chromatography and extrusion. Colloids Surf. A Physicochem. Eng. Asp..

[13] Wang X., Huang B., Liu X., Zhan P. (2016). Discovery of bioactive molecules from CuAAC click-chemistry-based combinatorial libraries. Drug Discov. Today.

[14] Yazdi M.K., Sajadi S.M., Seidi F., Rabiee N., Fatahi Y., Rabiee M., Dominic C.D.M., Zarrintaj P., Formela K., Saeb M.R. (2022). Clickable Polysaccharides for Biomedical Applications: A Comprehensive Review. Prog. Polym. Sci..

[15] Discher B.M., Won Y.Y., Ege D.S., Lee J.C.M., Bates F.S., Discher D.E., Hammer D.A. (1999). Polymersomes: Tough vesicles made from diblock copolymers. Science.

[16] Chandrawati R., Caruso F. (2012). Biomimetic liposome- and polymersome-based multicompartmentalized assemblies. Langmuir.

[17] Bermudez H., Brannan A.K., Hammer D.A., Bates F.S., Discher D.E. (2002). Molecular weight dependence of polymersome membrane structure, elasticity, and stability. Macromolecules.

[18] Cevc G., Marsh D. (1987). Phospholipid Bilayers Physical Principles and Models.

[19] Discher D.E., Ahmed F. (2006). Polymersomes. Annu. Rev. Biomed. Eng..

[20] Aranda-Espinoza H., Bermudez H., Bates F.S., Discher D.E. (2001). Electromechanical limits of polymersomes. Phys. Rev. Lett..

[21] Discher D.E., Eisenberg A. (2002). Polymer vesicles. Science.

[22] Photos P.J., Bacakova L., Discher B., Bates F.S., Discher D.E. (2003). Polymer vesicles in vivo: Correlations with PEG molecular weight. J. Control. Release.

[23] Alves A., Silva A.M., Moreira J., Nunes C., Reis S., Pinto M., Cidade H., Rodrigues F., Ferreira D., Costa P.C. (2024). Polymersomes for Sustained Delivery of a Chalcone Derivative Targeting Glioblastoma Cells. Brain Sci..

[24] Alves A., Silva A.M., Nunes C., Cravo S., Reis S., Pinto M., Sousa E., Rodrigues F., Ferreira D., Costa P.C. (2024). The Synthesis and Characterization of a Delivery System Based on Polymersomes and a Xanthone with Inhibitory Activity in Glioblastoma. Life.

[25] Ghasemiyeh P., Mohammadi-Samani S. (2018). Solid lipid nanoparticles and nanostructured lipid carriers as novel drug delivery systems: Applications, advantages and disadvantages. Res. Pharm. Sci..

[26] Kammari R., Das N.G., Das S.K. (2017). Nanoparticulate Systems for Therapeutic and Diagnostic Applications. Emerging Nanotechnologies for Diagnostics, Drug Delivery and Medical Devices.

[27] Aibani N., Khan T.N., Callan B. (2020). Liposome mimicking polymersomes; A comparative study of the merits of polymersomes in terms of formulation and stability. Int. J. Pharm. X.

[28] Matsumura Y., Hamaguchi T., Ura T., Muro K., Yamada Y., Shimada Y., Shirao K., Okusaka T., Ueno H., Ikeda M. (2004). Phase I clinical trial and pharmacokinetic evaluation of NK911, a micelle-encapsulated doxorubicin. Br. J. Cancer.

[29] Danson S., Ferry D., Alakhov V., Margison J., Kerr D., Jowle D., Brampton M., Halbert G., Ranson M. (2004). Phase I dose escalation and pharmacokinetic study of pluronic polymer-bound doxorubicin (SP1049C) in patients with advanced cancer. Br. J. Cancer.

[30] Chen H.-T., Neerman M.F., Parrish A.R., Simanek E.E. (2004). Cytotoxicity, Hemolysis, and Acute in Vivo Toxicity of Dendrimers Based on Melamine, Candidate Vehicles for Drug Delivery. J. Am. Chem. Soc..

[31] Derfus A.M., Chan W.C.W., Bhatia S.N. (2004). Probing the Cytotoxicity of Semiconductor Quantum Dots. Nano Lett..

[32] Bose S., Khare R.A., Moldenaers P. (2010). Assessing the strengths and weaknesses of various types of pre-treatments of carbon nanotubes on the properties of polymer/carbon nanotubes composites: A critical review. Polymer.

[33] Ferdous Z., Nemmar A. (2020). Health Impact of Silver Nanoparticles: A Review of the Biodistribution and Toxicity Following Various Routes of Exposure. Int. J. Mol. Sci..

[34] Pham Q.T., Ngo G.L., Nguyen X.A., Nguyen C.T., Ledoux-Rak I., Lai N.D. (2022). Direct Synthesis of Gold Nanoparticles in Polymer Matrix. Polymers.

[35] Kaur J., Saxena M., Rishi N. (2021). An Overview of Recent Advances in Biomedical Applications of Click Chemistry. Bioconjugate Chem..

[36] Matoori S., Leroux J.-C. (2020). Twenty-five years of polymersomes: Lost in translation?. Mater. Horiz..

[37] Fonseca M., Jarak I., Victor F., Domingues C., Veiga F., Figueiras A. (2024). Polymersomes as the Next Attractive Generation of Drug Delivery Systems: Definition, Synthesis and Applications. Materials.

[38] LoPresti C., Lomas H., Massignani M., Smart T., Battaglia G. (2009). Polymersomes: Nature inspired nanometer sized compartments. J. Mater. Chem..

[39] Guan L., Rizzello L., Battaglia G. (2015). Polymersomes and their applications in cancer delivery and therapy. Nanomedicine.

[40] Sharma A.K., Prasher P., Aljabali A.A., Mishra V., Gandhi H., Kumar S., Mutalik S., Chellappan D.K., Tambuwala M.M., Dua K. (2020). Emerging era of “somes”: Polymersomes as versatile drug delivery carrier for cancer diagnostics and therapy. Drug Deliv. Transl. Res..

[41] Hu Y., Qiu L. (2019). Polymersomes: Preparation and characterization. Methods Mol. Biol..

[42] Rideau E., Dimova R., Schwille P., Wurm F.R., Landfester K. (2018). Liposomes and polymersomes: A comparative review towards cell mimicking. Chem. Soc. Rev..

[43] Pallavi P., Harini K., Gowtham P., Girigoswami K., Girigoswami A. (2022). Fabrication of Polymersomes: A Macromolecular Architecture in Nanotherapeutics. Chemistry.

[44] Toprakcioglul C., Dail L., Ansarifarl M.A., Stamm M., Motschmann H. (1993). Equilibrium and dynamic aspects of end-attached diblock and triblock copolymer chains. Prog. Colloid. Polym. Sci..

[45] Napoli A., Tirelli N., Wehrli E., Hubbell J.A. (2002). Lyotropic behavior in water of amphiphilic ABA triblock copolymers based on poly(propylene sulfide) and poly(ethylene glycol). Langmuir.

[46] Stoenescu R., Graff A., Meier W. (2004). Asymmetric ABC-triblock copolymer membranes induce a directed insertion of membrane proteins. Macromol. Biosci..

[47] Wittemann A., Azzam T., Eisenberg A. (2007). Biocompatible polymer vesicles from biamphiphilic triblock copolymers and their interaction with bovine serum albumin. Langmuir.

[48] Walther A., Müller A.H.E. (2008). Janus particles. Soft Matter.

[49] Sommerdijk N.A.J.M., Holder S.J., Hiorns R.C., Jones R.G., Nolte R.J.M. (2000). Self-assembled structures from an amphiphilic multiblock copolymer containing rigid semiconductor segments. Macromolecules.

[50] Brannan A.K., Bates F.S. (2004). ABCA tetrablock copolymer vesicles. Macromolecules.

[51] Opsteen J.A., van Hest J.C.M. (2005). Modular synthesis of block copolymers via cycloaddition of terminal azide and alkyne functionalized polymers. Chem. Commun..

[52] Van Dongen S.F.M., Nallani M., Schoffelen S., Cornelissen J.J.L.M., Nolte R.J.M., van Hest J.C.M. (2008). A block copolymer for functionalisation of polymersome surfaces. Macromol. Rapid Commun..

[53] Van Dongen S.F.M., Nallani M., Cornelissen J.J.L.M., Nolte R.J.M., van Hest J.C.M. (2009). A Three-Enzyme Cascade Reaction through Positional Assembly of Enzymesin a Polymersome Nanoreactor. Chem. Eur. J..

[54] Binder W.H., Sachsenhofer R. (2008). Polymersome/silica capsules by ’click’-chemistry. Macromol. Rapid Commun..

[55] Kumar Upadhyay K., Le Meins J.F., Misra A., Voisin P., Bouchaud V., Ibarboure E., Schatz C., Lecommandoux S. (2009). Biomimetic doxorubicin loaded polymersomes from hyaluronan-block- poly(γ-benzyl glutamate) copolymers. Biomacromolecules.

[56] Shahriari M., Taghdisi S.M., Abnous K., Ramezani M., Alibolandi M. (2021). Self-targeted polymersomal co-formulation of doxorubicin, camptothecin and FOXM1 aptamer for efficient treatment of non-small cell lung cancer. J. Control. Release.

[57] Yin H., Kang H.C., Huh K.M., Bae Y.H. (2012). Biocompatible, pH-sensitive AB2 miktoarm polymer-based polymersomes: Preparation, characterization, and acidic pH-activated nanostructural transformation. J. Mater. Chem..

[58] Gaitzsch J., Chudasama V., Morecroft E., Messager L., Battaglia G. (2016). Synthesis of an Amphiphilic Miktoarm Star Terpolymer for Self-Assembly into Patchy Polymersomes. ACS Macro Lett..

[59] Khoee S., Hashemi A., Molavipordanjani S. (2018). Synthesis and characterization of IUdR loaded PEG/PCL/PEG polymersome in mixed DCM/DMF solvent: Experimental and molecular dynamics insights into the role of solvent composition and star architecture in drug dispersion and diffusion. Eur. J. Pharm. Sci..

[60] Rijpkema S.J., Langens S.G.H.A., van der Kolk M.R., Gavriel K., Toebes B.J., Wilson D.A. (2020). Modular Approach to the Functionalization of Polymersomes. Biomacromolecules.

[61] Malinge J., Allain C., Galmiche L., Miomandre F., Audebert P. (2011). Preparation, Photophysical, Electrochemical, and Sensing Properties of Luminescent Tetrazine-Doped Silica Nanoparticles. Chem. Mater..

[62] Barker I.A., Hall D.J., Hansell C.F., du Prez F.E., O’Reilly R.K., Dove A.P. (2011). Tetrazine-Norbornene Click Reactions to Functionalize Degradable Polymers Derived from Lactide. Macromol. Rapid Commun..

[63] Blackman M.L., Royzen M., Fox J.M. (2008). Tetrazine Ligation: Fast Bioconjugation Based on Inverse-Electron-Demand Diels−Alder Reactivity. J. Am. Chem. Soc..

[64] Penelas M.J., Soler-Illia G.J.A.A., Levi V., Bordoni A.V., Wolosiuk A. (2019). Click-based thiol-ene photografting of COOH groups to SiO2 nanoparticles: Strategies comparison. Colloids Surf. A Physicochem. Eng. Asp..

[65] Ruizendaal L., Pujari S.P., Gevaerts V., Paulusse J.M.J., Zuilhof H. (2011). Biofunctional Silicon Nanoparticles by Means of Thiol-Ene Click Chemistry. Chem. Asian J..

[66] Hoyle C.E., Bowman C.N. (2010). Thiol-Ene Click Chemistry. Angew. Chem. Int. Ed..

[67] Toebes B.J., Abdelmohsen L.K.E.A., Wilson D.A. (2018). Enzyme-driven biodegradable nanomotor based on tubular-shaped polymeric vesicles. Polym. Chem..

[68] Von Maltzahn G., Ren Y., Park J.-H., Min D.-H., Kotamraju V.R., Jayakumar J., Fogal V., Sailor M.J., Ruoslahti E., Bhatia S.N. (2008). In Vivo Tumor Cell Targeting with “Click” Nanoparticles. Bioconjug. Chem..

[69] Debets M.F., van der Doelen C.W.J., Rutjes F.P.J.T., van Delft F.L. (2010). Azide: A Unique Dipole for Metal-Free Bioorthogonal Ligations. ChemBioChem.

[70] Castillo R.R., Hernández-Escobar D., Gómez-Graña S., Vallet-Regí M. (2018). Reversible Nanogate System for Mesoporous Silica Nanoparticles Based on Diels-Alder Adducts. Chem. A Eur. J..

[71] Jarre G., Liang Y., Betz P., Lang D., Krueger A. (2011). Playing the surface game—Diels–Alder reactions on diamond nanoparticles. Chem. Commun..

[72] Shi M., Wosnick J.H., Ho K., Keating A., Shoichet M.S. (2007). Immuno-Polymeric Nanoparticles by Diels–Alder Chemistry. Angew. Chem. Int. Ed..

[73] Pawar P.V., Gohil S.V., Jain J.P., Kumar N. (2013). Functionalized polymersomes for biomedical applications. Polym. Chem..

[74] Pangburn T.O., Bates F.S., Kokkoli E. (2012). Polymersomes functionalized via “click” chemistry with the fibronectin mimetic peptides PR-b and GRGDSP for targeted delivery to cells with different levels of *α*5*β*1 expression. Soft Matter.

[75] Van Oers M.C.M., Abdelmohsen L.K.E.A., Rutjes F.P.J.T., van Hest J.C.M. (2014). Aqueous asymmetric cyclopropanation reactions in polymersome membranes. Chem. Commun..

[76] Van Oers M.C.M., Veldmate W.S., van Hest J.C.M., Rutjes F.P.J.T. (2015). Aqueous asymmetric aldol reactions in polymersome membranes. Polym. Chem..

[77] Eissa A.M., Smith M.J.P., Kubilis A., Mosely J.A., Cameron N.R. (2013). Polymersome-forming amphiphilic glycosylated polymers: Synthesis and characterization. J. Polym. Sci. A Polym. Chem..

[78] Opsteen J.A., Brinkhuis R.P., Teeuwen R.L.M., Löwik D.W.P.M., van Hest J.C.M. (2007). “Clickable” polymersomes. Chem. Commun..

[79] Li B., Martin A.L., Gillies E.R. (2007). Multivalent polymer vesicles via surface functionalization. Chem. Commun..

[80] Amos R.C., Nazemi A., Bonduelle C.V., Gillies E.R. (2012). Tuning polymersome surfaces: Functionalization with dendritic groups. Soft Matter.

[81] Nazemi A., Gillies E.R. (2013). Dendritic surface functionalization of nanomaterials- controlling properties and functions for biomedical applications. Braz. J. Pharm. Sci..

[82] Rein C., Nissen S., Grzelakowski M., Meldal M. (2016). Click-chemistry of polymersomes on nanoporous polymeric surfaces. J. Polym. Sci. A Polym. Chem..

[83] Karandish F., Mamnoon B., Feng L., Haldar M.K., Xia L., Gange K.N., You S., Choi Y., Sarkar K., Mallik S. (2018). Nucleus-Targeted, Echogenic Polymersomes for Delivering a Cancer Stemness Inhibitor to Pancreatic Cancer Cells. Biomacromolecules.

[84] Zhang Q., Ren H., Baker G.L. (2015). Synthesis and click chemistry of a new class of biodegradable polylactide towards tunable thermo-responsive biomaterials. Polym. Chem..

[85] Han Y., Yuan L., Li G., Huang L., Qin T., Chu F., Tang C. (2016). Renewable polymers from lignin via copper-free thermal click chemistry. Polymer.

[86] Li C., Finn M.G. (2006). Click chemistry in materials synthesis. II. Acid-swellable crosslinked polymers made by copper-catalyzed azide-alkyne cycloaddition. J. Polym. Sci. A Polym. Chem..

[87] Lang A.S., Neubig A., Sommer M., Thelakkat M. (2010). NMRP versus “Click” Chemistry for the Synthesis of Semiconductor Polymers Carrying Pendant Perylene Bisimides. Macromolecules.

[88] Gakiya-Teruya M., Palomino-Marcelo L., Pierce S., Angeles-Boza A.M., Krishna V., Rodriguez-Reyes J.C.F. (2020). Enhanced antimicrobial activity of silver nanoparticles conjugated with synthetic peptide by click chemistry. J. Nano. Res..

[89] Zou L., Shi Y., Cao X., Gan W., Wang X., Graff R.W., Hu D., Gao H. (2016). Synthesis of acid-degradable hyperbranched polymers by chain-growth CuAAC polymerization of an AB 3 monomer. Polym. Chem..

[90] Kita-Tokarczyk K., Grumelard J., Haefele T., Meier W. (2005). Block copolymer vesicles—Using concepts from polymer chemistry to mimic biomembranes. Polymer.

[91] Lee J.S., Feijen J. (2012). Polymersomes for drug delivery: Design, formation and characterization. J. Control. Release.

[92] Krishnamoorthy B., Karanam V., Chellan V.R., Siram K., Natarajan T.S., Gregory M. (2014). Polymersomes as an effective drug delivery system for glioma—A review. J. Drug Target..

[93] Striegel A.M., Yau W.W., Kirkland J.J., Bly D.D. (2009). Modern Size-Exclusion Liquid Chromatography.

[94] Chang R. Physical Chemistry for the Biosciences 2005. https://books.google.pt/books?id=PNH1fHj5Tw0C&printsec=frontcover&redir_esc=y#v=onepage&q&f=false.

[95] Skoog D., Holler F., Crouch S. Principles of Instrumental Analysis 1985. https://books.google.pt/books/about/Principles_of_Instrumental_Analysis.html?id=D13EDQAAQBAJ&redir_esc=y.

[96] Lathe G.H., Ruthven C.R. (1955). The Separation of Substances on the Basis of their Molecular Weights, Using Columns of Starch and Water. Biochem. J..

